# Risk factors and model construction for early neurological deterioration in patients with intracerebral hemorrhage

**DOI:** 10.3389/fneur.2025.1663347

**Published:** 2025-10-08

**Authors:** Heng Zhou, Dapeng Dai, Kang Xie, Aimin Li

**Affiliations:** ^1^Department of Neurosurgery, Institute of Neuroscience, Lianyungang Clinical College of Nanjing Medical University, The First People’s Hospital of Lianyungang, Lianyungang, China; ^2^Department of Neurosurgery, Institute of Neuroscience, The First People's Hospital of Lianyungang, The Affiliated Lianyungang Hospital of Xuzhou Medical University, Lianyungang, China

**Keywords:** intracerebral hemorrhage, early neurological deterioration, risk factors, predictive model, END

## Abstract

**Objective:**

To investigate the risk factors for early neurological deterioration (END) in patients with spontaneous intracerebral hemorrhage (ICH), construct a predictive model, and evaluate its predictive efficacy.

**Methods:**

We retrospectively selected 450 ICH patients admitted to the First People’s Hospital of Lianyungang from June 2023 to September 2024. The patients were randomly divided into a training set (315 patients) and a validation set (135 patients) at a 7:3 ratio. In the training set, patients were categorized into END group (*n* = 66) and non-END group (*n* = 249) based on the criteria of a decrease in GCS score by ≥2 points or an increase in NIHSS score by ≥4 points within 72 h of admission. We compared the general data, laboratory test results, and imaging features between the two groups. We used LASSO regression and multivariate logistic regression analysis to identify the independent risk factors for END in ICH patients. A nomogram model for predicting END in ICH patients was constructed using the R language rms package and applied to the validation set to assess the model’s predictive ability and accuracy by drawing ROC curves, calibration curves, and decision curve analysis (DCA) curves.

**Results:**

In the training set, there were significant differences between the END and non-END groups in terms of age, admission systolic blood pressure, admission GCS score, admission NIHSS score, serum potassium, serum calcium, blood glucose, homocysteine (Hcy), white blood cell count (WBC), C-reactive protein (CRP), neutrophil-to-lymphocyte ratio (NLR), intraventricular hemorrhage (IVH), blend sign, midline shift, hematoma expansion (HE), and initial hematoma volume (*p* < 0.05). The results of the LASSO regression and multivariate logistic regression analysis showed that the independent risk factors for END in ICH patients included age, WBC, Hcy, HE, blend sign, and admission systolic blood pressure. The area under the ROC curve (AUC) for predicting END in the training and validation sets were 0.909 and 0.831, respectively. The Hosmer-Lemeshow goodness-of-fit test showed that the model had good calibration (*p* = 0.550 for the training set and *p* = 0.368 for the validation set). The DCA curves in the training and validation sets indicated that the model had good clinical utility.

**Conclusion:**

Age, WBC, Hcy, HE, blend sign, and admission systolic blood pressure are independent risk factors for END in ICH patients. The nomogram model established based on these parameters can effectively predict END and provide a reference for clinical decision-making.

## 1 Introduction

Spontaneous intracerebral hemorrhage (ICH) refers to intracranial bleeding caused by the rupture of large or small arteries, veins, or capillaries within the skull in the absence of trauma. It is currently the second leading cause of fatal stroke globally, with a 30-day mortality rate as high as 35–52% (1–3). Despite early therapeutic measures such as dehydration to reduce intracranial pressure, temperature regulation, blood glucose stabilization, hematoma evacuation, and epilepsy prevention, only 12–39% of patients have a favorable prognosis (4). Early neurologic deterioration (END) is a common clinical complication of ICH, defined as persistent neurologic worsening occurring within 72 h after ICH onset. It is primarily characterized by decreased level of consciousness, new or worsening motor deficits, and impaired language function. END occurs in approximately 21–26% of ICH patients and is an important factor influencing poor prognosis (5, 6). However, the pathogenesis of END is not yet fully understood, and there is no consensus on its related risk factors, early diagnosis, or treatment strategies. Therefore, this study aims to conduct a retrospective analysis to identify the key risk factors for END, construct a nomogram model, and perform internal validation to assess the model’s efficacy. This will provide a reference for identifying high-risk populations for END, facilitating early intervention and improving patient outcomes.

## 2 Materials and methods

### 2.1 Study population

We selected 450 ICH patients admitted to the Department of Neurosurgery of the First People’s Hospital of Lianyungang from June 2023 to September 2024. The patients were divided into a training group and a validation group at a 7:3 ratio, with 315 patients in the training group and 135 patients in the validation group.

Inclusion criteria were as follows: (1) time from onset to admission <24 h; (2) Meeting the diagnostic criteria for ICH and confirmed by imaging studies at our hospital (7); (3) age ≥18 years; (4) time from onset to completion of laboratory and imaging examinations <24 h; (5) complete medical records, necessary laboratory tests, and imaging examination results.

Exclusion criteria included: (1) cerebral amyloid angiopathy or secondary ICH (e.g., trauma, aneurysm, arteriovenous malformation, hemorrhagic transformation of ischemic stroke, intracranial tumor, vasculitis, or other medical conditions); (2) poor neurological status (e.g., decerebrate coma, loss of brainstem function) or severe coagulopathy or dysfunction/failure of other vital organs (e.g., cardiac function class IV, uremia, liver failure); (3) patients who underwent emergency surgery immediately after admission; (4) patients with a history of anticoagulant or antiplatelet therapy before onset; (5) patients with incomplete clinical data or loss to follow-up. All patients were treated according to the 2022 Guideline for the Management of Patients With Spontaneous Hemorrhage during hospitalization (7). This study was approved by the Ethics Committee of the First People’s Hospital of Lianyungang (approval number: KY-20241017006-01).

The work has been reported in line with the STROCSS criteria.

### 2.2 Patient grouping

Within 30 min of admission, the Glasgow Coma Scale (GCS) and the National Institutes of Health Stroke Scale (NIHSS) were administered for the first time. Patients were re-evaluated within 72 h of admission. To ensure the objectivity and consistency of the evaluation results, each patient was assessed independently by two senior neurosurgeons. In case of inconsistent scores, a third senior physician was consulted until a consensus was reached. Patients were classified into the early neurological deterioration (END) group if their GCS score decreased by ≥2 points or their NIHSS score increased by ≥4 points within 72 h compared to admission scores; the remaining patients were assigned to the non-END group.

### 2.3 Data collection

#### 2.3.1 Baseline data

Baseline data were collected upon admission, including gender, age, body mass index (BMI), smoking history, drinking history, medical history of cerebral infarction, ICH, coronary heart disease, hypertension, diabetes, admission systolic blood pressure (SBP) and diastolic blood pressure (DBP), admission NIHSS score and GCS score.

#### 2.3.2 Laboratory tests

All laboratory indicators were obtained from the first venous blood sample within 24 h of admission, including serum potassium, serum sodium, serum calcium, total cholesterol (TC),triglycerides (TG), high-density lipoprotein cholesterol (HDL-C), low-density lipoprotein cholesterol (LDL-C), serum glucose, fibrinogen (FIB), international normalized ratio (INR), D-dimer, white blood cell count (WBC), C-reactive protein (CRP), homocysteine (Hcy), hemoglobin (Hb), platelet count (Plt), creatinine (Cr), blood urea nitrogen (BUN), and neutrophil-to-lymphocyte ratio (NLR).

#### 2.3.3 Imaging examinations

Non-contrast head CT was performed immediately upon admission to determine the location of the hemorrhage, whether the hematoma had ruptured into the ventricles, and whether there was midline shift. The hematoma volume was calculated using the formula by Saito (V = ABC/2, where A is the longest diameter of the hematoma in the axial plane, B is the longest diameter perpendicular to A in the same plane, and C is the number of visible hemorrhagic layers × slice thickness) (8). Head CT was repeated within 24 h. If the hematoma volume increased by >33% or the absolute volume change was >6 mL compared to the initial scan, it was defined as hematoma expansion (HE) (9). The “blend sign” was defined as a visible boundary between adjacent low- and high-density areas that were not completely enclosed and had a CT value difference of ≥18 HU (10). The “island sign” was defined as three or more round, discrete small hematomas completely separated from the main hematoma, or four or more budding small hematomas connected to the main hematoma (11). The “satellite sign” was defined as a patchy hematoma with a maximum transverse diameter <10 mm, separated from the main hematoma by a distance of 1–20 mm (12).

### 2.4 Statistical analysis

The statistical analysis in this study was conducted using R software (version 4.4.2) and SPSS 27.0. The 450 participants were randomly divided into a training group (*n* = 315) and a validation group (*n* = 135) at a 7:3 ratio. In the training group, the normal distribution of continuous data was tested. Normally distributed continuous data were expressed as mean ± standard deviation ($\bar{x}$±s), and comparisons between the two groups were made using the independent samples t-test. Non-normally distributed continuous data were expressed as median and interquartile range *M* (*P*_25_, *P*_75_), and the Mann–Whitney *U* test was used for comparison. Categorical data were expressed as percentages (%) and compared using the *χ^2^* test. We identified independent risk factors for END in ICH patients via LASSO and multivariate logistic regression analyses and constructed a nomogram. The model’s performance was validated by the validation group. Its goodness-of-fit was assessed by the Hosmer-Lemeshow test, with *p* > 0.05 indicating good calibration. Model performance was evaluated by ROC curves (AUC, sensitivity, specificity), and clinical utility by decision curve analysis (DCA). *p* < 0.05 was considered statistically significant.

## 3 Results

### 3.1 Comparison of general data

In the training group, a total of 315 patients were included, of whom 66 (20.95%) experienced END and 249 (79.05%) did not. The age, admission systolic blood pressure (SBP), and admission NIHSS scores of patients in the END group were all significantly higher than those in the non-END group (all *p* < 0.05). In contrast, the admission GCS scores (*p* < 0.05) was significantly lower in the END group. No significant differences were observed between the two groups in terms of body mass index (BMI), smoking history, drinking history, medical history of diabetes, hypertension, coronary heart disease, cerebral infarction, ICH, admission DBP (*p* > 0.05) (Table 1).

**Table 1:** Comparison of general data between END and non-END groups.

Factor	END group (*n* = 66)	Non-END group (*n* = 249)	Statistic value	*p*-value
Age (years)	68 (63.75,75.25)	61 (53,70)	4.423^b^	<0.001
Gender (*n*)			1.690^c^	0.194
Male	35 (53.0)	154 (61.8)		
Female	31 (47.0)	95 (38.2)		
BMI	26.28 ± 4.82	25.68 ± 4.05	−1.017^a^	0.122
Smoking history (*n*)			0.665^c^	0.415
Yes	17 (25.8)	77 (30.9)		
No	49 (74.2)	172 (69.1)		
Drinking history (*n*)			0.156^c^	0.693
Yes	21 (31.8)	73 (29.3)		
No	45 (68.2)	176 (70.7)		
History of cerebral infarction (*n*)			0.757^c^	0.384
Yes	13 (19.7)	38 (15.3)		
No	47 (75.8)	211 (84.7)		
History of ICH (*n*)			0.009^c^	0.923
Yes	5 (7.6)	18 (7.2)		
No	61 (92.4)	231 (92.8)		
History of coronary heart disease (*n*)			0.311^c^	0.577
Yes	4 (6.1)	11 (4.4)		
No	62 (93.9)	238 (95.6)		
History of hypertension (*n*)			2.377^c^	0.123
Yes	62 (93.9)	217 (87.1)		
No	4 (6.1)	32 (12.9)		
History of diabetes (*n*)			0.160^c^	0.690
Yes	10 (15.2)	33 (13.3)		
No	56 (84.6)	216 (86.7)		
Admission SBP (mmHg)	170.15 ± 21.06	156.72 ± 19.33	-4.923^a^	<0.001
Admission DBP (mmHg)	95.06 ± 14.28	93.31 ± 13.54	-0.922^a^	0.357
Admission GCS score	11 (8,13)	14 (11,14)	-5.596^b^	<0.001
Admission NIHSS score	15 (10,20)	10 (5,15)	5.221^b^	<0.001

^a^*t*-value, ^b^*Z*-value, ^c^*χ*^2^-value.

### 3.2 Comparison of laboratory data

Patients in the END group had significantly higher levels of serum potassium, serum calcium, blood glucose, WBC, Hcy, CRP, and NLR compared to those in the non-END group (all *p* < 0.05), with statistically significant differences. No significant differences were found between the two groups in BUN, Cr, TC, TG, HDL-C, LDL-C, Hb, Plt, INR, or FIB (all *p* > 0.05) (Table 2).

**Table 2:** Comparison of laboratory data between END and non-END groups.

Factor	END group (*n* = 66)	Non-END group (*n* = 249)	Statistic value	*p*-value
Serum potassium (mmol/L)	3.67 ± 0.47	3.85 ± 0.40	3.099^a^	0.002
Serum sodium (mmol/L)	138.30 (136.03, 139.98)	137.80 (135.70, 139.50)	1.237^b^	0.216
Serum calcium (mmol/L)	2.22 (2.13, 2.27)	2.27 (2.21, 2.32)	−3.764^b^	<0.001
Serum glucose (mmol/L)	7.23 (5.99, 9.36)	6.30 (5.29, 7.57)	3.649^b^	<0.001
Hcy (umol/L)	14.30 (10.45, 17.75)	10.60 (8.40,13.25)	5.271^b^	<0.001
BUN (mmol/L)	5.68 (4.35, 7.47)	5.37 (4.17, 6.30)	1.822^b^	0.068
Cr (umol/L)	66.80 (57.83, 74.83)	62.60 (53.40, 71.30)	1.819^b^	0.069
TC (mmol/L)	4.41 (3.62, 5.32)	4.49 (3.96, 5.30)	−0.509^b^	0.611
TG (mmol/L)	0.88 (0.60, 1.32)	0.93 (0.66, 1.40)	−0.571^b^	0.568
HDL-C (mmol/L)	1.20 (0.98, 1.37)	1.20 (1.00, 1.38)	−0.513^b^	0.608
LDL-C (mmol/L)	2.74 (2.21, 3.29)	2.86 (2.38, 3.45)	−1.307^b^	0.191
WBC (×10^9/L)	10.12 (8.62, 13.79)	8.32 (6.61, 10.05)	5.211^b^	<0.001
Hb (g/L)	135.0 (126.5, 143.5)	135.0 (123.0, 144.0)	0.125^b^	0.901
Plt (×10^9/L)	215.32 ± 62.16	213.56 ± 58.46	−0.215^a^	0.830
CRP (mg/L)	8.77 (2.65, 20.68)	2.60 (1.06, 5.60)	5.233^b^	<0.001
NLR	10.60 (6.64, 18.00)	5.57 (3.55, 9.03)	6.658^b^	<0.001
INR	1.05 (1.01, 1.07)	1.05 (1.00, 1.09)	−0.253^b^	0.800
FIB (g/L)	2.95 (2.38, 3.54)	2.95 (2.38, 3.54)	0.406^b^	0.685

^a^*t*-value, ^b^*Z*-value, ^c^*χ*^2^-value.

### 3.3 Comparison of imaging data

The initial hematoma volume, proportion of IVH, midline shift, HE, and blend sign were significantly higher in the END group compared to the non-END group (all *p* < 0.05), with statistically significant differences. No significant differences were observed between the two groups in the specific location of bleeding, the presence of the island sign or satellite sign (*p* > 0.05) (Table 3).

**Table 3:** Comparison of imaging data between END and non-END groups.

Factor	END group (*n* = 66)	Non-END group (*n* = 249)	Statistic value	*p*-value
Cerebellar hemorrhage (*n*)			0.610^c^	0.435
Yes	7 (10.6)	19 (7.6)		
No	59 (89.4)	230 (92.4)		
Basal ganglia hemorrhage (*n*)			0.190^c^	0.663
Yes	29 (43.9)	102 (41.0)		
No	37 (56.1)	147 (59.0)		
Thalamic hemorrhage (*n*)			0.030^c^	0.862
Yes	19 (28.8)	69 (27.7)		
No	47 (71.2)	180 (72.3)		
Lobar hemorrhage (*n*)			0.441^c^	0.507
Yes	16 (24.2)	51 (20.5)		
No	50 (75.8)	198 (79.5)		
Brainstem hemorrhage (*n*)			0.111^c^	0.739
Yes	5 (7.6)	16 (6.4)		
No	61 (92.4)	233 (93.6)		
IVH (*n*)			11.700^c^	<0.001
Yes	31 (47.0)	63 (25.3)		
No	35 (53.0)	186 (74.7)		
Blend sign (*n*)			12.255^c^	<0.001
Yes	27 (40.9)	50 (20.1)		
No	39 (59.1)	199 (79.9)		
Satellite sign (*n*)			0.169^c^	0.681
Yes	11 (16.7)	47 (18.9)		
No	55 (83.3)	202 (81.1)		
Island sign (*n*)			1.668^c^	0.197
Yes	11 (16.7)	27 (10.8)		
No	55 (83.3)	222 (89.2)		
Midline shift (*n*)			4.613^c^	0.032
Yes	10 (15.2)	17 (6.8)		
No	56 (84.8)	232 (93.2)		
HE (*n*)			68.881^c^	<0.001
Yes	30 (68.2)	14 (5.6)		
No	36 (54.5)	235 (94.4)		
Initial hematoma volume (ml)	24.06 (11.46, 35.31)	8.82 (4.70, 17.72)	6.202^b^	<0.001

^a^*t*-value, ^b^*Z*-value, ^c^*χ*^2^-value.

### 3.4 Data screening

**Figure 1:**
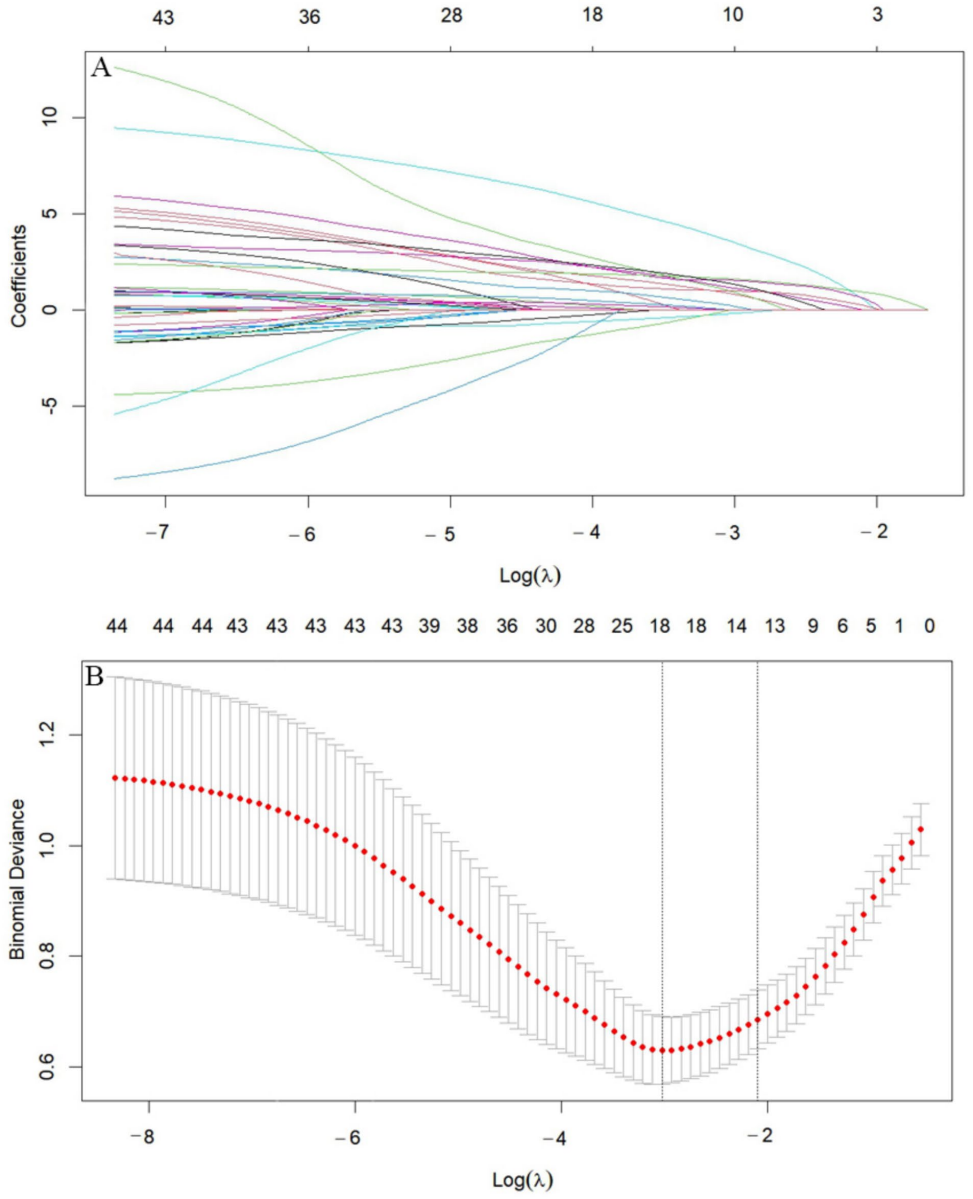
LASSO diagram. **(A)** Cross-validation curve. **(B)** Feature shrinkage path. **(A)** Includes 44 variables corresponding to 44 lines in different colors. Each curve represents the trajectory of change for each independent variable. The vertical axis indicates the coefficient value, while the horizontal axis below shows log(*λ*). As the parameter log(λ) increases, the vertical axis continuously converges to zero. **(B)** Shows the X-axis representing the logarithm of the penalty coefficient log λ, with the Y-axis indicating the likelihood deviation. A smaller Y-value indicates a better fit of the equation.

We performed LASSO regression on 315 patients in the training set and 44 variables (Figure 1). This identified 13 potential risk factors for END after ICH: age, admission GCS score, serum calcium, blood glucose, Hcy, WBC, CRP, NLR, IVH, initial hematoma volume, HE, admission SBP, and the blend sign. The variable coding is shown in Table 4. Multivariate logistic regression analysis of these 13 variables is shown in Table 5. The results showed that age (OR = 1.042, 95% CI = 1.006–1.080), admission SBP (OR = 1.031, 95% CI = 1.009–1.053), Hcy (OR = 1.226, 95% CI = 1.113–1.350), WBC (OR = 1.170, 95% CI = 1.016–1.347), HE (OR = 13.688, 95% CI = 4.408–42.505), and the blend sign (OR = 3.084, 95% CI = 1.220–7.797) were independent risk factors for END after ICH. Collinearity diagnostics are shown in Table 6. The tolerance was greater than 0.1 and the variance inflation factor (VIF) was less than 10, indicating no multicollinearity among these six variables.

**Table 4:** Variable coding.

Variable	Coding
Age	Original value input
Admission SBP	Original value input
Admission GCS score	Original value input
Serum calcium	Original value input
Serum glucose	Original value input
Hcy	Original value input
WBC	Original value input
CRP	Original value input
NLR	Original value input
IVH	No IVH = 0, IVH = 1
Blend sign	No blend sign = 0, Blend sign present = 1
HE	No HE = 0, HE = 1
Initial hematoma volume	Original value input
END	No END = 0, END present = 1

**Table 5:** Multivariate logistic regression analysis results for END in ICH patients.

Variable	B	SE	Wald	OR	95% CI	*p*-value
Age	0.041	0.018	5.137	1.042	1.006–1.080	0.023
Admission SBP	0.031	0.011	7.962	1.031	1.009–1.053	0.005
Admission GCS score	−0.075	0.080	0.880	0.928	0.794–1.085	0.348
Serum calcium	−3.359	2.223	2.282	0.035	0.000–2.716	0.131
Serum glucose	0.115	0.081	1.997	1.122	0.957–1.315	0.158
Hcy	0.204	0.049	17.030	1.226	1.113–1.350	<0.001
WBC	0.157	0.072	4.748	1.170	1.016–1.347	0.029
CRP	0.009	0.007	1.728	1.009	0.996–1.022	0.189
NLR	0.042	0.038	1.186	1.043	0.967–1.124	0.276
IVH	−0.673	0.441	2.326	0.510	0.215–1.212	0.127
Blend sign	1.126	0.473	5.665	3.084	1.220–7.797	0.017
HE	2.616	0.578	20.482	13.688	4.408–42.505	<0.001
Initial hematoma volume	0.019	0.015	1.473	1.019	0.989–1.050	0.225
Constant	−7.574	6.050	1.568	-	-	0.211

B, regression coefficient; SE, standard error; Wald, Wald test; OR, odds ratio.

**Table 6:** Collinearity diagnosis for END in ICH patients.

Variable	Tolerance	VIF
Age	0.960	1.042
Hcy	0.913	1.095
WBC	0.907	1.102
Admission SBP	0.962	1.039
Blend sign	0.967	1.034
HE	0.942	1.061

### 3.5 Nomogram model construction and validation

**Figure 2:**
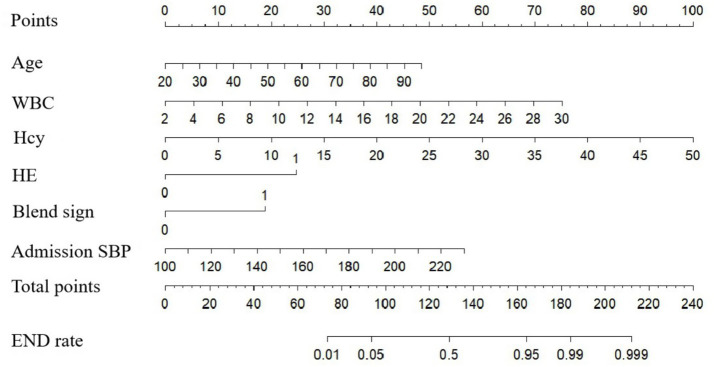
Risk nomogram model for END in ICH patients.

A nomogram model for predicting END in ICH patients was constructed based on the predictive variables derived from multivariate logistic regression analysis (age, admission SBP, Hcy, WBC, HE and the blend sign) (Figure 2). The nomogram assigns scores to each independent risk factor according to its weight in predicting END. Medical staff can sum the scores of each risk factor for a patient on the nomogram, and the total score corresponds to the predicted probability of END occurrence.

**Figure 3:**
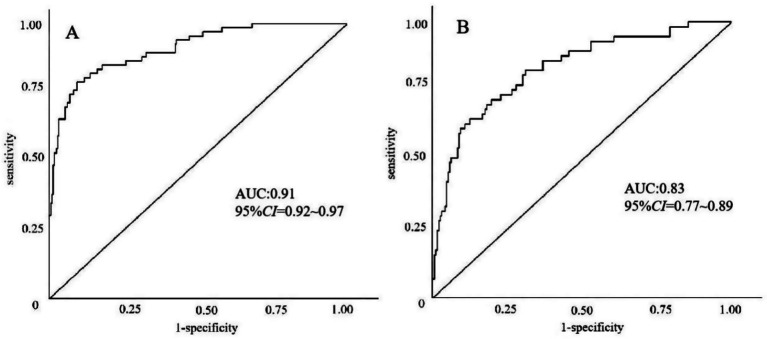
ROC curves for the nomogram model predicting END in ICH patients. **(A)** Training set. **(B)** Validation set.

**Figure 4:**
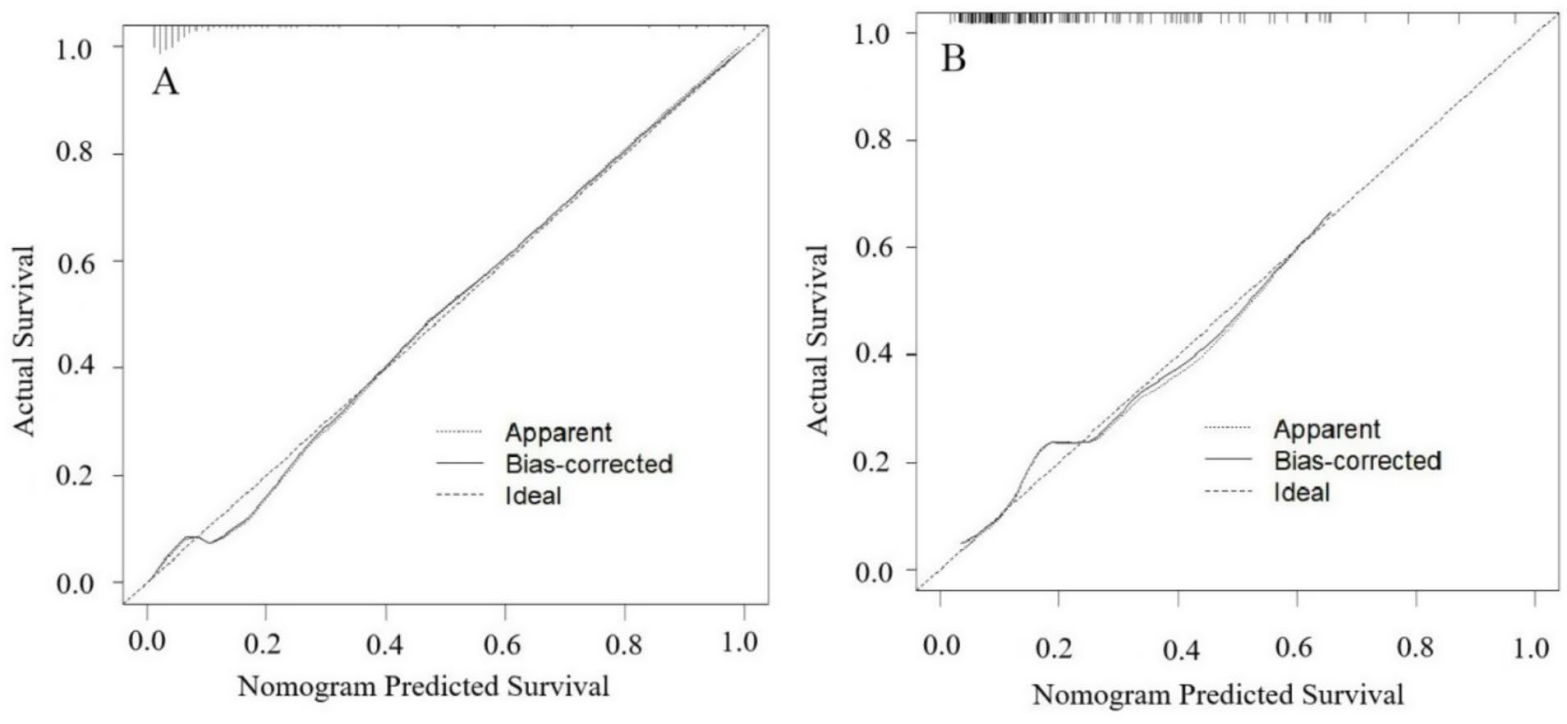
Calibration curves for the nomogram model predicting END in ICH patients. **(A)** Training set. **(B)** Validation set. The apparent line represents the calibration performance of the model on the current dataset. The bias-corrected line uses statistical methods to correct the model, estimating its more realistic calibration performance on new data.

**Figure 5:**
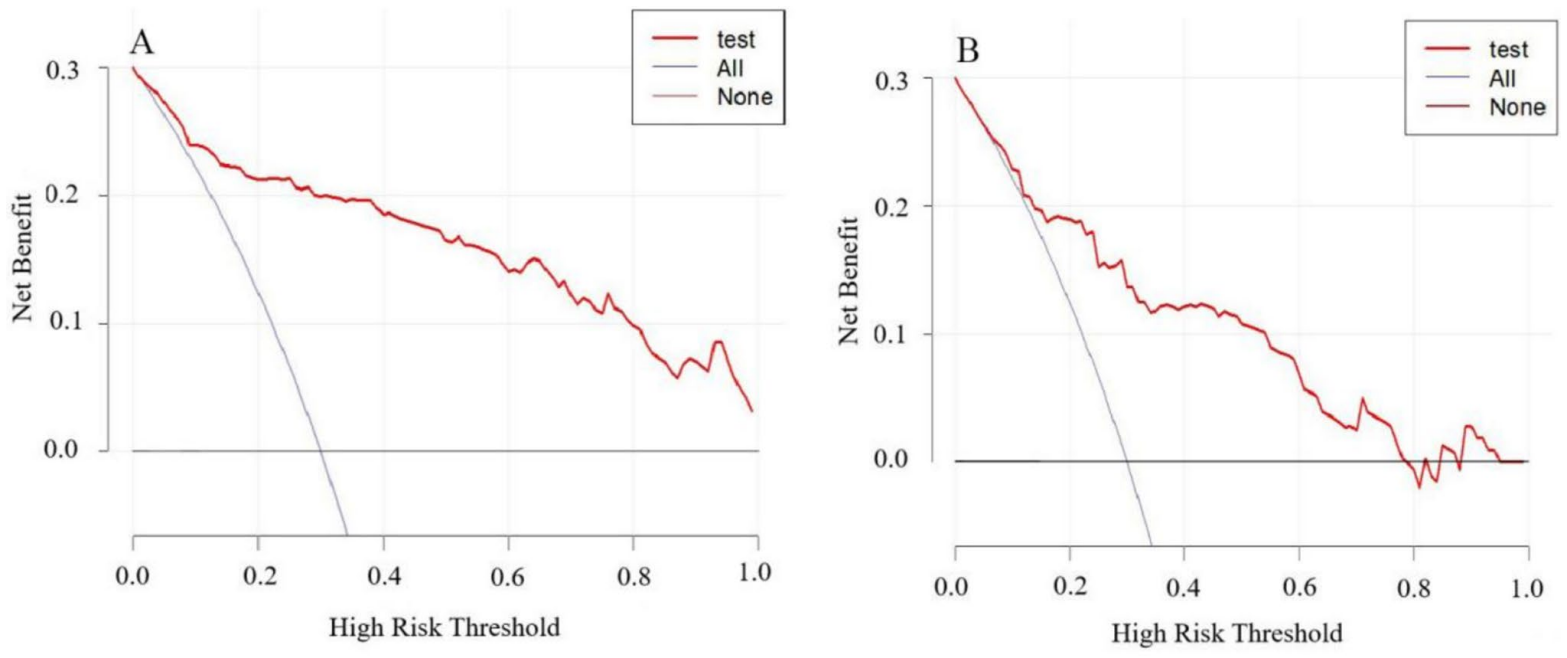
DCA curves for the nomogram model predicting END in ICH patients. **(A)** Training set. **(B)** Validation set.

The model’s performance was tested using the validation group. There were no statistically significant differences between the training and validation groups for any variables (all *p* > 0.05) (Table 7). ROC curves for the training and validation groups are shown in Figure 3. The AUC values were 0.909 (95% CI = 0.867–0.951) and 0.831 (95% CI = 0.769–0.893), respectively, indicating good discrimination. Calibration curves for the training and validation groups are shown in Figure 4. The model’s predicted probabilities closely matched the actual probabilities. Hosmer-Lemeshow goodness-of-fit tests showed good model calibration (training group: *X^2^* = 6.879, *p* = 0.550; validation group: *X^2^* = 8.703, *p* = 0.368). DCA curves for the training and validation groups are shown in Figure 5. The x-axis represents the threshold probability and the y-axis represents net benefit. The calibration curves were above the All and None lines, indicating good clinical utility of the model for predicting END.

**Table 7:** Differences between the training set and testing set.

Factor	Training set (*n* = 315)	Validation set (*n* = 135)	Statistic value	*p*-value
Age (years)	64 (54,71)	62 (53,72)	−0.770^b^	0.441
Gender (*n*)			0.770^c^	0.380
Male	189 (60.0)	75 (55.6)		
Female	126 (40.0)	60 (44.4)		
BMI	25.39 (23.23, 28.76)	25.39 (23.44, 28.31)	−0.195^b^	0.845
Smoking history (*n*)			0.131^c^	0.718
Yes	94 (29.80)	38 (28.10)		
No	221 (70.20)	97 (71.90)		
Drinking history (*n*)			0.617^c^	0.432
Yes	98 (31.1)	37 (27.4)		
No	217 (68.9)	98 (72.6)		
History of cerebral infarction (*n*)			2.329^c^	0.127
Yes	51 (16.2)	30 (22.2)		
No	264 (83.8)	105 (77.8)		
History of ICH (*n*)			1.179^c^	0.277
Yes	23 (7.3)	14 (10.4)		
No	292 (92.7)	121 (89.6)		
History of coronary heart disease (*n*)			0.264^c^	0.607
Yes	15 (4.8)	8 (5.9)		
No	300 (95.2)	127 (94.1)		
History of hypertension (*n*)			0.325^c^	0.569
Yes	279 (88.6)	117 (86.7)		
No	36 (11.4)	18 (13.3)		
History of diabetes (*n*)			0.014^c^	0.905
Yes	43 (13.7)	19 (14.1)		
No	272 (86.3)	116 (85.9)		
Admission SBP (mmHg)	159.54 ± 20.42	162.39 ± 21.05	−1.347^a^	0.179
Admission DBP (mmHg)	94 (85,102)	95 (85,103)	0.537^b^	0.591
Admission GCS score	13.00 (10.75,14.00)	14.00 (11.00,14.00)	1.612^b^	0.107
Admission NIHSS score	10 (6,17)	10 (6,15)	−0.849^b^	0.396
Serum potassium (mmol/L)	3.81 ± 0.42	3.73 ± 0.40	1.912^a^	0.057
Serum sodium (mmol/L)	140.31 ± 4.98	139.58 ± 4.97	−1.274^a^	0.204
Serum calcium (mmol/L)	2.26 (2.20, 2.32)	2.24 (2.17, 2.30)	−1.497^b^	0.134
Serum glucose (mmol/L)	6.43 (5.43, 8.16)	6.44 (5.44, 7.94)	−0.164^b^	0.870
Hcy (μmol/L)	11.3 (8.98, 14.53)	10.60 (8.40, 13.70)	−1.153^b^	0.249
BUN (mmol/L)	5.40 (4.20, 6.47)	5.30 (4.10, 7.00)	0.285^b^	0.776
Cr (μmol/L)	64.80 (55.13, 73.40)	65.00 (57.00, 72.40)	0.040^b^	0.968
TC (mmol/L)	4.49 (3.90,5.30)	4.61 (3.98, 5.33)	1.007^b^	0.314
TG (mmol/L)	0.91 (0.65,1.40)	1.04 (0.74, 1.45)	1.740^b^	0.082
HDL-C (mmol/L)	1.19 (0.99,1.37)	1.21 (1.03, 1.41)	1.024^b^	0.306
LDL-C (mmol/L)	2.84 (2.34, 3.41)	2.93 (2.50, 3.60)	1.441^b^	0.150
WBC (×10^9/L)	8.64 (6.92, 10.51)	8.95 (7.14, 11.20)	1.043^b^	0.297
Hb (g/L)	135 (124, 144)	133 (124, 145)	−0.104^b^	0.917
Plt (×10^9/L)	212 (175, 247)	212 (163, 255)	−0.371^b^	0.710
CRP (mg/L)	3.25 (1.16, 8.31)	2.83 (0.84, 7.46)	−0.562^b^	0.574
NLR	6.42 (3.91, 10.84)	6.66 (4.21, 10.64)	0.500^b^	0.617
INR	1.05 (1.00, 1.09)	1.03 (0.99, 1.08)	−1.387^b^	0.165
FIB (g/L)	2.90 (2.41, 3.47)	2.98 (2.46, 3.55)	0.916^b^	0.360
Cerebellar hemorrhage (*n*)			0.226^c^	0.635
Yes	26 (8.3)	13 (9.6)		
No	289 (91.7)	122 (90.4)		
Basal ganglia hemorrhage (*n*)			1.304^c^	0.254
Yes	131 (41.6)	64 (47.4)		
No	184 (58.4)	71 (52.6)		
Thalamic hemorrhage (*n*)			1.595^c^	0.207
Yes	88 (27.9)	30 (22.2)		
No	227 (72.1)	105 (77.8)		
Lobar hemorrhage (*n*)			1.473^c^	0.225
Yes	67 (21.3)	22 (16.3)		
No	248 (78.7)	113 (83.7)		
Brainstem hemorrhage (*n*)			0.355^c^	0.551
Yes	21 (6.7)	7 (5.2)		
No	294 (93.3)	128 (94.8)		
IVH (*n*)			0.013^c^	0.911
Yes	94 (29.8)	41 (30.4)		
No	221 (70.2)	94 (69.6)		
Blend sign (*n*)			0.111^c^	0.739
Yes	77 (24.4)	35 (25.9)		
No	238 (75.6)	100 (74.1)		
Satellite sign (*n*)			2.960^c^	0.085
Yes	58 (18.4)	16 (11.9)		
No	257 (81.6)	119 (88.1)		
Island sign (*n*)			0.265^c^	0.607
Yes	38 (12.1)	14 (10.4)		
No	277 (87.9)	121 (89.6)		
Midline shift (*n*)			0.922^c^	0.337
Yes	27 (8.6)	8 (5.9)		
No	288 (91.4)	127 (94.1)		
HE (*n*)			0.409^c^	0.522
Yes	44 (14.0)	22 (16.3)		
No	271 (86.0)	113 (83.7)		
Initial hematoma volume (ml)	10.66 (5.34, 22.64)	8.24 (4.33, 18.06)	−1.680^b^	0.093

^a^*t*-value, ^b^*Z*-value, ^c^*χ*^2^-value.

## 4 Discussion

Early neurological deterioration (END) is a severe complication of spontaneous intracerebral hemorrhage (ICH), imposing a heavy burden on patients, their families, and society. The pathogenesis of END involves multiple factors, including ferroptosis of neurons, inflammatory cascades, collateral circulation impairment, and abnormal increases in intracranial pressure (13). The incidence of END in this study was 20.26%, which is consistent with previous reports (5). Identifying risk factors for END and recognizing high-risk patients early to implement effective therapeutic measures are of great significance for improving patient prognosis and are the focus of current research.

Clinical studies on END in ICH patients have been reported, although with varying conclusions. However, risk factors such as age, HE, and IVH have been widely confirmed (14–20), which is consistent with the findings of this study. Patients of advanced age are at a higher risk of developing ICH and often have multiple underlying diseases, such as coronary heart disease and diabetes, which can lead to structural changes in the cerebral blood vessels. Meanwhile, with increasing age, brain tissue gradually atrophies and becomes more susceptible to injury, both of which contribute to the occurrence of END (21, 22). Additionally, HE can compress brain tissue, obstruct local blood circulation, induce cerebral edema, and further deteriorate neurological function, potentially leading to herniation in severe cases (23, 24). Hematomas that rupture into the ventricles can block the fourth ventricle and cerebral aqueduct, causing obstruction of cerebrospinal fluid circulation, increased intracranial pressure, hydrocephalus, and worsening of neuronal injury, ultimately leading to END (25).

The Glasgow Coma Scale (GCS) and the National Institutes of Health Stroke Scale (NIHSS) are two important tools used in clinical practice to assess patients’ neurological status. The GCS primarily evaluates the level of consciousness, while the NIHSS assesses the degree of neurological deficit. Both can quantify the severity of brain injury and guide early interventions. Low GCS scores or high NIHSS scores indicate severe consciousness impairment, poor neurological function, and a tendency for the condition to worsen (26, 27). In this study, both scores showed statistically significant differences between the END and non-END groups, but they were not independent risk factors for END. The reason may be that newly identified risk factors, such as Hcy and the blend sign, are more sensitive in predicting END, thus overshadowing the predictive role of traditional scores. Moreover, initial hematoma volume was also not an independent risk factor for END in this study, suggesting that a large hematoma volume is more closely related to immediate coma or even death after disease onset, whereas END refers to the continuous deterioration of the patient’s condition after admission. In comparison, HE, which causes a “second hit” to the patient, is the intrinsic factor driving the occurrence of END.

Hypertension is the most common cause of ICH (28). In this study, high systolic blood pressure was found to be an independent risk factor for END, even though most ICH patients have hypertension. However, the admission SBP in the END group was significantly higher than that in the non-END group, indicating that a dramatic increase in blood pressure at the time of onset can still be fatal to the brain. Despite no differences in admission DBP between the two groups, this may be related to the fact that patients received guideline-recommended antihypertensive treatment after admission, achieving similar blood pressure levels. A study by Qureshi AI (29) found that for patients with admission SBP ≥ 220 mmHg, those who underwent standard antihypertensive treatment to 140–179 mmHg within 24 h had a lower incidence of END compared to those who received intensive antihypertensive treatment to 110–139 mmHg (15.5% vs. 6.8%; *p* = 0.04). For hypertensive patients, strictly controlling blood pressure fluctuations, setting stepwise antihypertensive targets, and balancing cerebral perfusion with the risk of rebleeding are key measures to prevent the occurrence of END (30, 31).

Hyperglycemia can damage multiple systems in the human body, including the nervous, cardiovascular, and immune systems (32–34), and this study also confirmed that it is a risk factor for END. The reasons may include: (1) Hyperglycemia induces the activation of astrocytes, which release pro-inflammatory factors that promote the aggregation of white blood cells (WBCs) and exacerbate neuro-inflammatory responses (35); (2) Hyperglycemia intensifies oxidative stress reactions at the hemorrhagic site, disrupts normal neuronal metabolism, causes secondary damage, and further destroys the structure and function of neurons (36); (3) Aquaporin-4 (AQP4) is a key channel in glial cells that regulates water balance. After ICH, it can promote the expulsion of water molecules from cells to alleviate edema. However, hyperglycemia inhibits the expression of AQP4 in damaged brain cells, thereby worsening cerebral edema and leading to END (37). A meta-analysis by Jiao X (38) found that glucose variability is more accurate in predicting poor outcomes in ICH patients than fasting blood glucose (FBG). However, there are currently no reports on the relationship between glucose variability and END, which is also a limitation of this study and needs further exploration in the future.

After ICH, WBCs migrate into the brain, aggregate around the hemorrhagic site, and release a large number of inflammatory mediators (such as elastase and myeloperoxidase), inducing local inflammatory cascades and damaging normal brain tissue, which is an important pathological mechanism for the occurrence of END (39). In recent years, some studies have found that inflammatory cell ratios, such as the neutrophil-to-lymphocyte ratio (NLR), a reliable and easily obtained novel inflammatory marker, also show good performance in predicting END (40, 41), and this study also confirmed this conclusion. Neutrophils, as the first WBCs to infiltrate brain tissue from peripheral blood after ICH, increase capillary permeability and disrupt the blood–brain barrier through various inflammatory reactions, promoting the infiltration of inflammatory cells (such as monocytes and T lymphocytes) into brain parenchyma and causing cerebral edema (42, 43). Lymphocytes are the main immune regulatory cells in the body, and a decrease in their number indicates an immunosuppressive state. After ICH, the body activates the hypothalamic–pituitary–adrenal axis and the sympathetic-adrenal medullary axis, causing an endogenous stress response. This leads to the massive secretion of glucocorticoids and catecholamines, causing lymphocyte apoptosis and inducing immunosuppression in the body. This can lead to stroke-related pneumonia, urinary tract infections, and other nosocomial infectious diseases, causing symptoms such as persistent high fever, increased metabolism, and metabolic disturbances (such as lactic acidosis and electrolyte imbalances), which can deal a secondary blow to patients and worsen their condition (44). A study by Liesz et al. (45) found that the degree of lymphocyte count reduction is significantly positively correlated with the risk of infection in ICH patients. Both elevated neutrophils and reduced lymphocytes have been shown to be closely related to poor prognosis in ICH patients. NLR, by integrating these two indicators, can reflect the dynamic changes in the intensity of systemic inflammatory response and the degree of immune system damage after ICH (46–48), thereby effectively predicting END.

Hcy was confirmed as an independent risk factor for END after ICH in this study. Previous studies have mostly reported that Hcy is associated with END in ischemic stroke through promoting atherosclerosis (49, 50), with fewer reports on its relationship with ICH. High levels of Hcy can significantly upregulate the expression of matrix metalloproteinase-2 (MMP-2) and matrix metalloproteinase-9 (MMP-9) in vascular endothelial cells, specifically degrading key components of the basement membrane such as type IV collagen and laminin, leading to the rupture of elastic fibers in the arterial wall (51). Additionally, reactive oxygen species (ROS) generated during Hcy metabolism can upregulate the levels of pro-inflammatory factors (such as IL-6 and IL-8), induce inflammatory responses, weaken the repair capacity of the vascular wall, increase the risk of rupture, lead to HE, and thereby promote END (50, 52). Zhou et al. (53) found in a study of 69 ICH patients that patients with high Hcy (≥14.62 mmol/L) had a larger average bleeding volume than those with low Hcy (<14.62 mmol/L) (13.18 mL vs. 23.09 mL, *p* = 0.012). However, no correlation was found in patients with lobar or infratentorial ICH, which may be related to the small sample size included. A multicenter retrospective cohort study from the China Stroke Center Alliance, including 55,793 ICH patients, showed that high Hcy (≥15 mmol/L) is an independent risk factor for poor prognosis in ICH patients (OR = 1.06, 95% CI = 1.01–1.10, *p* = 0.010) (54). Supplementation with folic acid and vitamin B can effectively reduce Hcy levels in ICH patients, prevent vascular endothelial injury and oxidative stress reactions, and reduce the occurrence of HE, END, and poor prognosis, becoming a potential target for treating secondary damage in ICH (55).

Some specific CT signs can independently predict HE, such as the blend sign, satellite sign, and island sign (56). Meanwhile, HE is closely related to END, but few studies have reported the relationship between CT signs and END. This study found that the blend sign is independent predictors of END. The blend sign reflects the heterogeneity of hematoma density. Old coagulated bleeding, due to hemoglobin concentration and fibrin deposition, appears as high density on CT images, while active bleeding, containing liquid plasma, tends to appear as low density on CT. The presence of the blend sign indicates multiple foci of recurrent active bleeding in the skull and a longer duration of bleeding, leading to HE and driving the occurrence of END (57). The satellite sign reflects the morphological heterogeneity of the hematoma. Following ICH, the brain tissue surrounding the hematoma undergoes a series of pathological processes, including cytotoxic edema, ischemia–reperfusion injury, and disruption of the blood–brain barrier. These processes ultimately lead to continuous microvascular extravasation and the development of multiple small focal bleedings around the main hematoma. On CT imaging, this manifests as an irregular shape of the hematoma, with the satellite sign serving as a specific representation and a crucial indicator for predicting hematoma expansion (HE) and poor prognosis (58). However, in this study, the island sign and satellite sign were not a risk factor for END, which may be due to the small sample size included, leading to bias. More large-scale, multicenter, prospective studies are needed in the future to verify the application value of CT signs in the assessment of END.

In recent years, risk nomograms, as a type of visual predictive tool, have been widely used in medical research and practice. This study combined six indicators—age, admission SBP, Hcy, WBC, HE, the blend sign—to construct a nomogram predictive model for END after ICH. The validation results showed that the model has good discriminatory ability, calibration, and clinical utility. It can effectively help clinicians accurately identify high-risk populations for END, this enables clinicians to take targeted measures such as antihypertensive therapy, dehydration, hemostasis, and even surgery, thereby preventing the occurrence of END and improving the prognosis.

Although this study has preliminarily constructed a predictive model with clinical application value, there are still some limitations. For example, the data are from a single center, and the conclusions lack representativeness. The sample size is small, and the evidence is not very persuasive. As a retrospective study, there may be selection bias. The judgment of imaging signs may be subjective. Other variables that may affect END have not been included. In the future, multicenter validation studies should be carried out in regional medical centers to continuously optimize model parameters, establish a dynamic updating mechanism, and improve the model’s generalizability to ensure its widespread application in various populations.

## Data Availability

The original contributions presented in the study are included in the article/supplementary material, further inquiries can be directed to the corresponding authors.
